# University of Michigan

**Published:** 1875-07-01

**Authors:** 


UNIVERSITY OF MICHIGAN.—The Regents of the University of Michigan have finally accepted the act of the

Legislature of that State providing for a College of Homœopathy in connection with the Medical Department. The special school is to consist of two professors, one of Materia Medica and Therapeutics, the other of Practical Medicine. For instruction in all other branches the students are to attend the old Medical Department. It is reported that Dr. Sager, one of the oldest and most influential members of the regular medical faculty, has resigned. His chair was that of Obstetrics and Diseases of Women.

